# The Role of GaN in the Heterostructure WS_2_/GaN for SERS Applications

**DOI:** 10.3390/ma16083054

**Published:** 2023-04-12

**Authors:** Tsung-Shine Ko, En-Ting Lin, Yen-Teng Ho, Chen-An Deng

**Affiliations:** 1 Department of Electronic Engineering, National Changhua University of Education, No. 2, Shi-Da Road, Changhua 50074, Taiwan; ting19960105@gmail.com (E.-T.L.); chenan880110@gmail.com (C.-A.D.); 2 International College of Semiconductor Technology, National Yang Ming Chiao Tung University, Hsinchu 30010, Taiwan; chia500@nycu.edu.tw

**Keywords:** SERS, WS_2_, GaN, heterostructure, charge transfer

## Abstract

In the application of WS_2_ as a surface–enhanced Raman scattering (SERS) substrate, enhancing the charge transfer (CT) opportunity between WS_2_ and analyte is an important issue for SERS efficiency. In this study, we deposited few-layer WS_2_ (2–3 layers) on GaN and sapphire substrates with different bandgap characteristics to form heterojunctions using a chemical vapor deposition. Compared with sapphire, we found that using GaN as a substrate for WS_2_ can effectively enhance the SERS signal, with an enhancement factor of 6.45 × 10^4^ and a limit of detection of 5 × 10^−6^ M for probe molecule Rhodamine 6G according to SERS measurement. Analysis of Raman, Raman mapping, atomic force microscopy, and SERS mechanism revealed that The SERS efficiency increased despite the lower quality of the WS_2_ films on GaN compared to those on sapphire, as a result of the increased number of transition pathways present in the interface between WS_2_ and GaN. These carrier transition pathways could increase the opportunity for CT, thus enhancing the SERS signal. The WS_2_/GaN heterostructure proposed in this study can serve as a reference for enhancing SERS efficiency.

## 1. Introduction

Surface-enhanced Raman scattering (SERS) technique is a powerful tool for detecting and characterizing molecules at very low concentrations [1,2]. However, the SERS signal can still be weak for some analytes and in some experimental conditions. Improving the SERS signal would enable more sensitive detection and better characterization of molecules, which can have important applications in fields such as biomedicine, environmental monitoring, and materials science [3,4,5,6]. Additionally, a better understanding of the mechanism behind SERS enhancement is important for further optimizing the technique and developing new applications. The SERS effect is generally accepted to occur through the following two mechanisms: the electromagnetic mechanism (EM) and the chemical mechanism (CM). The use of noble metals in SERS methods is commonly adopted to achieve a significant enhancement in signal due to high electromagnetic fields around metallic structures [7,8] in the case of the EM effect. The metallic structure can provide a boost of several orders of magnitude to SERS signals. The CM effect, on the other hand, refers to the chemical interaction between the analyte molecule and the substrate [9,10]. Generally, the SERS signal of the CM effect is much lower than that of the EM effect. In recent years, several studies have demonstrated that 2D materials such as graphene, boron nitride, and molybdenum disulfide (MoS_2_) can be utilized as SERS substrates for biomedical detections [11,12]. 

Among 2D materials used for SERS measurements, tungsten disulfide (WS_2_) is a two–dimensional layered material that has recently attracted great interest in electronic and optoelectronic applications due to its high electron mobility, direct band gap, and strong absorption properties in the visible and near–infrared regions [13,14,15,16]. Recent research has also pointed out that the high sensitivity, good chemical stability, and excellent biocompatibility of WS_2_ make it highly promising for SERS applications in detecting probe molecules [17,18,19]. The mechanism behind the use of WS_2_ as a SERS substrate to enhance the SERS signal intensity of the analyte is attributed to a CM, where the exciton generated under light illumination undergoes a CT process between the energy levels of the analyte and WS_2_ at their interface, causing a significant enhancement of Raman signals achieved through charge transitions [20].

Compared to the noble metals such as Au and Ag, which are commonly used for enhancing the SERS signal through the electromagnetic mechanism, the enhancement effect of the SERS signal using CM of WS_2_ is weaker and limited [21]. Therefore, it is necessary to enhance the signal intensity by using nanostructures. However, the process of producing nanostructured WS_2_ is complex, and the size is difficult to control [22]. Therefore, we propose using a simple semiconductor heterostructure of WS_2_/GaN to effectively enhance the SERS signal. In this study, we utilized a metal-organic chemical vapor deposition (MOCVD) system to grow GaN on sapphire, followed by the deposition of a few-layer WS_2_ film on both GaN and sapphire substrates using a furnace with chemical vapor deposition (CVD) progress. After that, we compared the SERS performance of the two substrates using rhodamine (R6G) as the analyte. Our analysis, which included Raman spectroscopy, Raman mapping, atomic force microscopy (AFM), scanning electron microscopy (SEM), and X-ray photoelectron spectroscopy (XPS), showed that the quality of the WS_2_ films grown on GaN was inferior to those grown on sapphire. Despite this, we observed that using GaN as a substrate for WS_2_ can significantly enhance the SERS signal, with an enhancement factor of 6.45 × 10^4^ and a limit of detection of 5 × 10^−6^ M for the probe molecule R6G, as confirmed by SERS measurements. Through analysis of the CT mechanism, it can be inferred that an appropriate semiconductor heterostructure such as WS_2_/GaN can effectively provide more paths for carrier transition, making the effect of CT more intense and further enhancing the SERS signal intensity. A series of results could be expected to contribute to the development of using semiconductor heterostructures as SERS substrates.

## 2. Materials and Methods

### 2.1. Fabrication of WS_2_/GaN Heterostructure

A close–coupled showerhead MOCVD system was utilized to grow regular thin films of c–plane GaN on c–plane sapphire. A GaN nucleation layer with a thickness of around 30 nm was deposited by MOCVD at a growth temperature of 530 °C and a pressure of 600 mbar on c–plane sapphire substrates. The initial GaN layer was deposited at a growth temperature of 1030 °C and a pressure of 300 mbar. The temperature and pressure for the main GaN layer were set at 1050 °C and 150 mbar, respectively, with a V/III ratio of approximately 1200. The thickness of the GaN was approximately 2 μm. Afterward, the WS_2_ film was uniformly grown on both GaN and sapphire substrates positioned at the center of the furnace through the CVD process. The Ar/H_2_ carrier gas ratio was about 3:1 (150 sccm and 50 sccm). During growing few-layer WS_2_, the growth gas used was a mixture of H_2_S (10% to Ar) and WF_6_, with a flow ratio of H_2_S/WF_6_ equal to 200:1. 

### 2.2. Preparation of R6G Molecules

A solution of R6G (ACROS Organics, 99%, Geel, Belgium) in water with concentrations ranging from 10^−2^ to 10^−6^ M was prepared and dip–coated onto the WS_2_/GaN, WS_2_/sapphire, and sapphire substrates. The 10^−2^ M R6G solution was also placed onto a flat sapphire as a reference substrate, thereby allowing measurement of the EF. The specimens were subjected to ambient conditions and dried on a hot plate at 70 °C for 5 min. 

### 2.3. Characterizations

A confocal Raman microscopy system (Tokyo Instruments, Nanofinder 30, Tokyo, Japan) was used to perform Raman spectra and SERS analyses. The excitation was carried out by a He–Ne laser, and the laser power used was 0.1 mW for as–grown samples and 0.3 mW for the reference substrate. The laser spot size was adjusted to 3 μm using a microscope objective with a magnification of 100× and a numerical aperture of 0.9. The acquisition time for laser measurements was configured to 20 s, while the spectrometer employed a grating with a density of 300 lines per millimeter. The resolution of Raman spectrometer was about 0.5 cm^−1^. The spectrometer’s charge–coupled device was cooled to around −50 °C, utilizing a thermoelectric cooling chip to minimize measurement noise. Prior to measurement, the spectra were calibrated using the peak (520 cm^−1^) of the Si on the bulk Si substrate. The surface morphology of the samples was examined using an atomic force microscope (Veeco DI–3100, Plainview, NY, USA) through an AFM scan. SEM (FEI Helios 1200+, Hillsboro, OR, USA) revealed the morphologies and nanostructures of the as-grown samples. The chemical configurations of the samples were determined using an XPS instrument (ULVAC-PHI Phi V5000, Chigasaki, Japan) equipped with an Al Kα X-ray source and applied to the samples for analysis.

## 3. Results and Discussion

**Figure 1:**
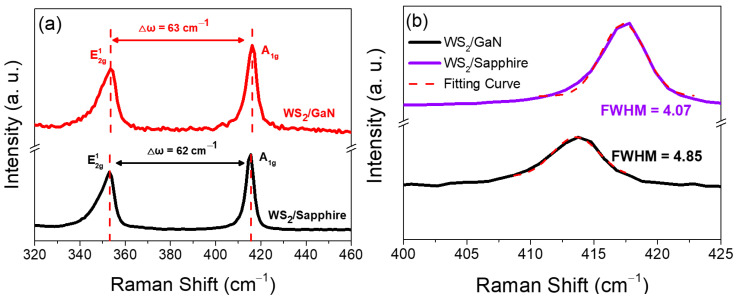
(**a**) Raman spectra of few-layer WS_2_ grown on GaN and sapphire. (**b**) Enlarged range of the A_1g_ peak in Figure 1a and corresponding Gaussian fitting result.

WS_2_ is a two-dimensional material in which the layers interact with each other through van der Waals forces [23]. Even though there is a bonding force between WS_2_ and the underlying GaN atoms, the resulting stress has a negligible effect on the arrangement of the lattice above WS_2_. Thus, we were unable to use X-ray diffraction to analyze the aforementioned information and instead were only able to further analyze the thickness of WS_2_ on GaN and sapphire using Raman spectroscopy. The molecule vibration modes of the WS_2_ grown on GaN and sapphire substrates under identical CVD conditions were examined through Raman spectroscopy analysis. The Raman spectra depicted in Figure 1a reveal the typical E^1^_2g_ and A_1g_ vibration mode peaks of WS_2_ for both WS_2_/GaN and WS_2_/sapphire samples [24], indicating successful deposition of the WS_2_ layer on the respective substrates. A widely recognized phenomenon in the field is that the difference in wavenumber between the two vibration modes E^1^_2g_ and A_1g_ of WS_2_ exhibits a decreasing trend with the reduction in WS_2_ thickness from bulk to few-layered structures [25,26]. Based on the observed difference in wavenumber of approximately 63 cm^−1^ for the WS_2_/GaN sample and 62 cm^−1^ for the WS_2_/sapphire sample, it can be inferred that the WS_2_ thin film formed on both substrates has a thickness ranging from 2 to 3 layers. On the other hand, it has been reported by McCreary et al. that the quality of WS_2_ thin film can be determined by observing the full–width at half–maximum (FWHM) of the Raman A_1g_ peak [27]. Therefore, the A_1g_ peaks of the two samples mentioned above were fitted with Gaussian curves to obtain accurate FWHM values. As shown in Figure 1b, the FWHM of the WS_2_ A_1g_ peak grown on sapphire was 4.07 cm^−1^, while that on GaN was approximately 4.85 cm^−1^. This result suggests that the WS_2_ grown on sapphire has better thin film quality compared to that grown on GaN. The reason for this will be discussed later. 

**Figure 2:**
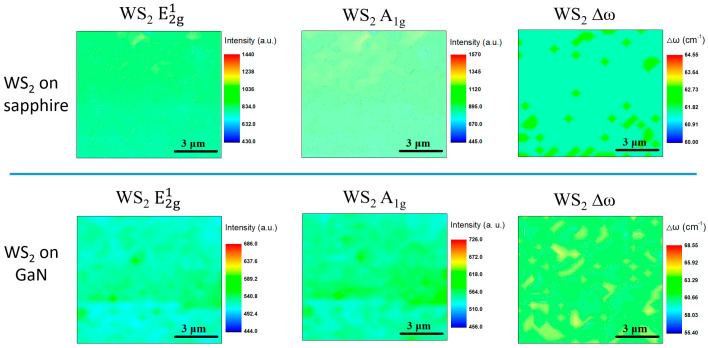
Raman mapping results of E^1^_2g_, A_1g_, and Δω for WS_2_/sapphire and WS_2_/GaN.

**Figure 3:**
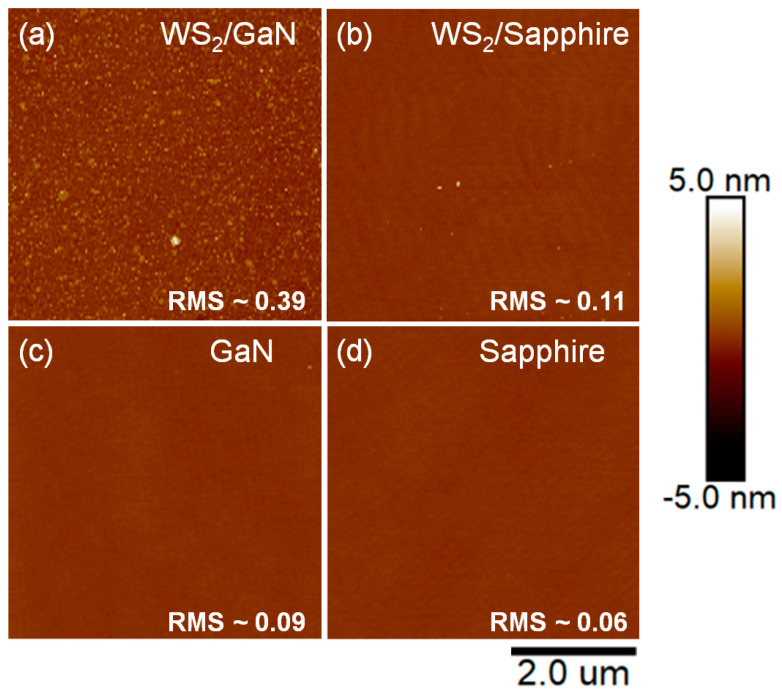
AFM images of different samples: (**a**) WS_2_/GaN, (**b**) WS_2_/sapphire, (**c**) GaN, and (**d**) sapphire.

In order to further compare the uniformity of WS_2_ grown on GaN and sapphire substrates in terms of Raman results, we further utilized Raman mapping to obtain the spatial distribution of the two peaks E^1^_2g_ and A_1g_, as well as the wavenumber difference (Δω) between the two peaks. As shown in Figure 2, when WS_2_ was grown on sapphire, the uniformity of both E^1^_2g_ and A_1g_ peaks was significantly higher than that of GaN. In addition, the results of Δω also showed that the thickness uniformity was better when grown on sapphire. Overall, the thickness range of WS_2_ grown on GaN was larger, which also verified the results of Figure 1a. Furthermore, our AFM measurement results, shown in Figure 3a,b, indicate that the root mean square (RMS) values of surface roughness for WS_2_/GaN and WS_2_/sapphire are 0.39 and 0.11, respectively. This suggests that the surface of WS_2_/GaN is rougher than that of WS_2_/sapphire, consistent with the aforementioned Raman and Raman mapping results. The two AFM images in Figure 3c,d provide further evidence that the surface of GaN is not as flat as that of sapphire, as the former has undergone MOCVD epitaxial growth while the latter has been polished. The RMS roughness of GaN is approximately 0.09, while that of sapphire is 0.06. We also conducted additional SEM observations, as shown in Figure S2a,b in the Supplementary Materials. The WS_2_ grown on GaN appeared significantly rougher than the WS_2_ grown on sapphire. It can be observed that the SEM results are consistent with the AFM results. This explains why the subsequent growth of WS_2_ on GaN, in terms of surface uniformity and thickness uniformity, is inferior to that on sapphire. Another possible reason is that during the furnace CVD growth process, due to its higher surface activity compared to sapphire, GaN is more prone to adsorb some of the native contaminants in the furnace, such as O, C, and S atoms [28], which can affect the interface smoothness of the subsequently grown WS_2_ film. Therefore, it can be concluded that the WS_2_ grown on GaN exhibits inferior lattice quality and surface roughness compared to that grown on sapphire. Regarding this aspect, we provide XPS data and have a detailed discussion below.

**Figure 4:**
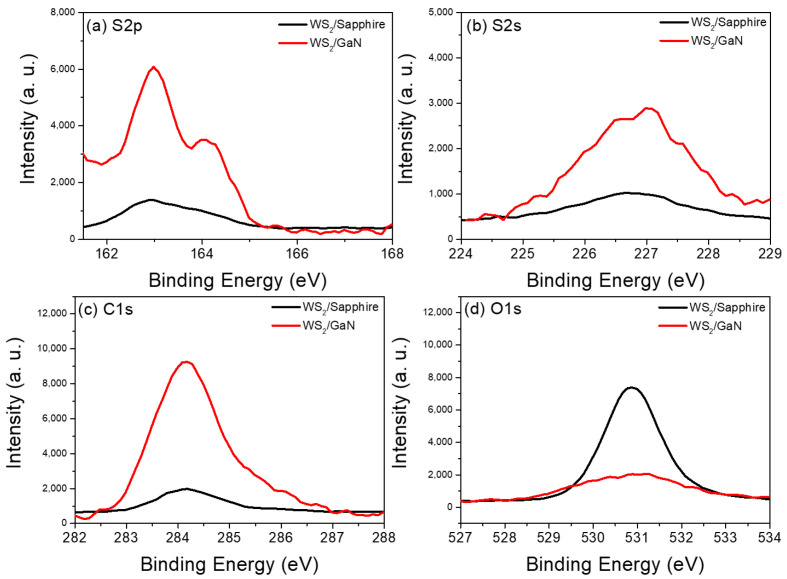
(**a**) S2p, (**b**) S2s, (**c**) C1s and (**d**) O1s core level XPS spectra of both WS_2_/GaN and WS_2_/sapphire.

To further understand the reason for the poor surface quality of WS_2_ grown on GaN, we performed XPS experiments to analyze the content of S, C, and O atoms. The results of XPS are shown below in Figure 4a–d. Based on the signals of (a) S2p, (b) S2s, and (c) C1s, it can be concluded that when WS_2_ was grown on GaN, both the amount of S and C atoms were higher than when it was grown on sapphire. This result is consistent with our AFM results mentioned above. Compared to sapphire, GaN is more prone to absorb S, C, and other atoms on its surface during the growth of WS_2_, which leads to poorer surface quality. On the other hand, as shown in Figure 4d, there are relatively more O atoms in the WS_2_ on the sapphire structure. It is well known that sapphire has a higher affinity for oxygen compared to GaN [29]. This is because sapphire has a more polar surface with a higher surface energy, making it easier for oxygen atoms to be absorbed or captured by the surface. In contrast, GaN has a nonpolar surface with lower surface energy, which makes it less likely for oxygen atoms to be captured or absorbed. Another possible reason for the higher concentration of O atoms could be the presence of O atoms in the sapphire substrate itself.

**Figure 5:**
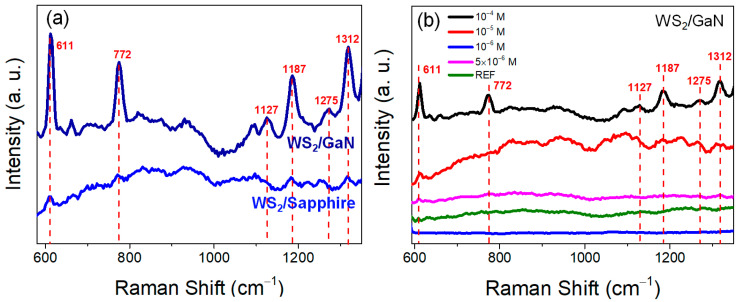
(**a**) Comparison of SERS results for measuring 10^−4^ M R6G using WS_2_/GaN and WS_2_/sapphire. (**b**) SERS results for different concentrations of R6G using WS_2_/GaN.

We conducted SERS measurements on these two types of SERS substrates using R6G of 10^−4^ M as the analyte, as shown in Figure 5a. The results indicate that under this range of wavenumbers, the main R6G SERS signals measured on WS_2_/GaN are significantly stronger than those on WS_2_/sapphire, with the peak at 611 cm^−1^ being particularly prominent [30]. Although WS_2_/sapphire has better film quality, WS_2_/GaN shows a more prominent performance as a SERS substrate. Moreover, we used this WS_2_/GaN heterostructure substrate to measure different concentrations of R6G solution, as shown in Figure 5b. It can be observed that a weak 611 cm^−1^ peak signal is still present at 5 × 10^−6^ M, but no R6G signal can be detected at 10^−6^ M, indicating that the LOD of WS_2_/GaN, in our case, can reach 5 × 10^−6^ M. Generally, the EF is an important metric for evaluating the performance of SERS because it quantifies the level of signal enhancement that occurs when analyte molecules are adsorbed onto either a semiconductor or plasmonic substrate. EF is calculated by comparing the Raman signal intensity of a given molecule on a plasmonic substrate with that of the same molecule in its bulk form. A high EF indicates that the plasmonic substrate provides a strong enhancement effect on the Raman signal of the analyte, leading to a more sensitive detection method. Therefore, the EF value is commonly used as a figure of merit to compare the performance of different SERS substrates and to optimize the SERS experimental conditions. According to the calculation formula of EF shown in the following equation [31]:

(1) $$\mathrm{EF}=\frac{I_{\mathrm{SERS}}}{I_{\mathrm{REF}}}\times \frac{N_{REF}}{N_{SERS}},$$

where *I* is the Raman spectral intensity and *N* is the number of molecules, respectively; SERS and REF denote the values obtained from WS_2_/GaN and the reference sample of the sapphire substrate, respectively. As the laser spot size and exposure time were constant for all measurements, the ratio of *N*_REF_/*N*_SERS_ was equivalent to the R6G concentration ratio for both samples. According to the above equation, it can be determined that the EF for the peak 611 cm^−1^ of R6G measured using the WS_2_/GaN SERS substrate can reach 6.45 × 10^4^. Therefore, in terms of overall SERS performance, WS_2_/GaN performs better than WS_2_/sapphire. In the following discussion, we will explore the advantages of using GaN in the WS_2_ heterostructure for SERS measurement based on our experimental results. We also conducted measurements on the relationship between the 611 cm^−1^ peak intensity and different R6G concentrations (ranging from 10^−4^ M to 5 × 10^−6^ M) for both samples. The results are presented in Figure S3 in the Supplementary Materials. It is apparent from the figure that the peak intensity WS_2_/GaN was consistently higher than that of WS_2_/sapphire in this R6G concentration range, indicating that our measurements were highly reliable.

**Figure 6:**
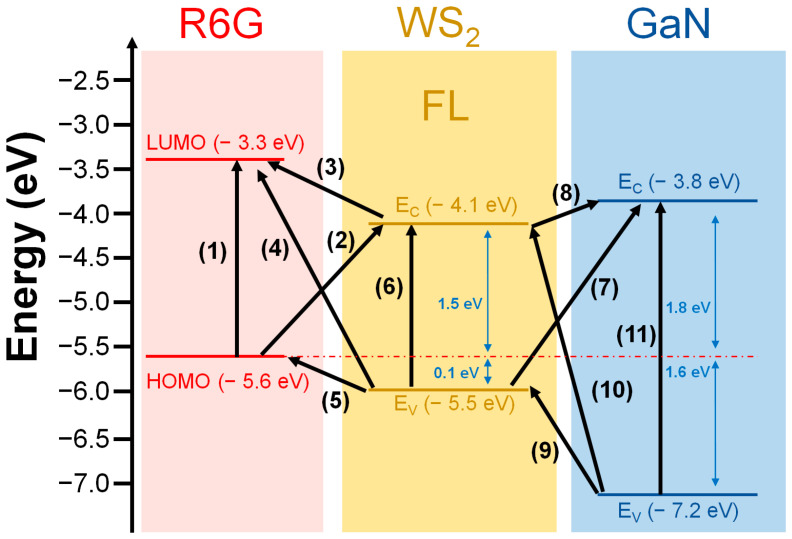
Schematic diagram of CT pathways between R6G molecules, few-layer WS_2_ and GaN.

In general, the enhancement mechanism of semiconductors as SERS substrates mainly comes from the CT between the photoexcited carriers and the analyte in the band gap of the semiconductor [32]. Lee et al. reported that WS_2_ demonstrates better SERS performance as the number of atomic layers decreases [33]. From a band perspective, our Raman results indicate that WS_2_ has a thickness of only about 2–3 atomic layers and a direct bandgap of about 1.6 eV [34]. The laser energy used in our experiments is approximately 1.96 eV, as shown in Figure 6, which can provide carrier transition pathways (2), (3), (5), and (6). The corresponding energy levels are the lowest unoccupied molecular orbital (LUMO) and highest occupied molecular orbital (HOMO) of R6G, as well as the conduction band edge energy level (E_c_) and valence band edge energy level (E_v_) of WS_2_. The energy differences between these pathways are all smaller than the energy of the laser, allowing WS_2_ to effectively enhance the SERS signal intensity of R6G. On the other hand, when GaN is introduced as a substrate for WS_2_ to form the WS_2_/GaN heterostructure, the Raman results in Figure S1 show that the laser intensity can penetrate through the thin WS_2_ film and measure the oscillation frequency signals E_2_ and A_1_ of GaN [35]. This demonstrates that the laser can also excite carriers in the interface between WS_2_ and GaN with energy differences smaller than the laser energy. Compared to sapphire, GaN has a bandgap of approximately 3.4 eV, which at the interface with 1.6 eV of WS_2_, can provide additional effective carrier transition pathways (7)–(9). This allows the excited electrons to have the opportunity to migrate to the interface between R6G and WS_2_, increasing the probability of CT and enhancing the SERS signal intensity. For the purpose of comparison, we have listed the various pathways and their corresponding energy levels in Table 1, along with the possibility of carrier transition. 

In addition to the transition pathways between the WS_2_/GaN heterostructure mentioned above that contribute to the strong SERS signal, other possibilities still need to be considered. Our AFM results show that the surface of WS_2_ grown on GaN is rougher than that grown on sapphire. Therefore, the higher roughness of WS_2_ on GaN also provides a larger contact area with the probe molecule R6G, which could enhance the SERS signal. Furthermore, in the above discussions, we mentioned that the native impurities O, C, and S in the furnace tube can be easily adsorbed onto the GaN surface, forming additional deep levels in the GaN bandgap. Therefore, these impurities may provide additional transition pathways for both WS_2_ and GaN, thereby increasing the SERS intensity. However, our current experimental results do not confirm the contribution of these reasons to the enhancement of the SERS signal. Therefore, in the future, we will design further experiments to analyze these possible factors.

## 4. Conclusions

In conclusion, we propose the use of GaN as a substrate for few-layer WS_2_ to form WS_2_/GaN heterostructures as SERS substrates and compare their thin film quality and SERS performance with WS_2_/sapphire. Through a series of experiments, including Raman, Raman mapping, AFM, and SERS mechanism investigation, our results showed that the quality of few-layer WS_2_ film grown on GaN was inferior to that on sapphire. However, due to the high surface roughness associated with high surface contact and the additional carrier transition paths in the WS_2_/GaN interface, WS_2_/GaN heterostructures could effectively enhance SERS signals in detecting the R6G target molecule compared to WS_2_/sapphire, with an EF value of 6.45 × 10^4^ and a LOD of 5 × 10^−6^ M. Therefore, our study suggests that designing appropriate semiconductor heterostructure SERS substrates for different target molecules can effectively increase the chance of CT and enhance SERS signals. 

## Figures and Tables

**Table 1:** Corresponding energy differences and probabilities of carrier transition pathways indicated in Figure 6.

**Pathways**	(1)	(2)	(3)	(4)	(5)	(6)	(7)	(8)	(9)	(10)	(11)
**ΔE (eV)**	2.3	1.5	0.8	2.2	0.1	1.4	1.7	0.3	1.7	3.1	3.4
**Possibility**	X	O	O	X	O	O	O	O	O	X	X

## Data Availability

Data is contained within the article or Supplementary Materials.

## Supplementary Materials

The following supporting information can be downloaded at: https://www.mdpi.com/article/10.3390/ma16083054/s1, Figure S1: Additional Raman results of WS_2_/GaN heterostructure. Figure S2: SEM images of different samples: (a) WS_2_/GaN, and (b) WS_2_/sapphire. Figure S3. A relationship between the Raman peak intensities at 611 cm^−1^ and the R6G concentrations (from 5 × 10^−6^ to 10^−4^ M).
