# Supplementary material for: The Role of GaN in the Heterostructure WS2/GaN for SERS Applications
Source: Materials (Basel). 2023 Apr 12;16(8):3054. doi: 10.3390/ma16083054 (PMC10143599; doi:10.3390/ma16083054)
Supplement: Supplementary file 1 [file materials-16-03054-s001.zip › materials-2305113-supplementary.pdf]

Supplementary materials

The role of GaN in the heterostructure WS_2/GaN for SERS applications

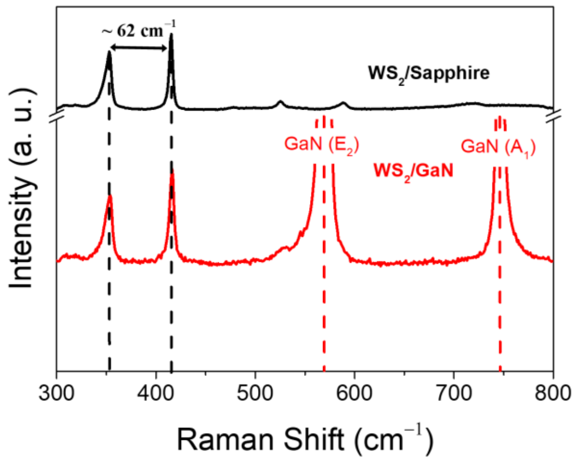

Figure S1. Additional Raman results of WS_2/GaN heterostructure.

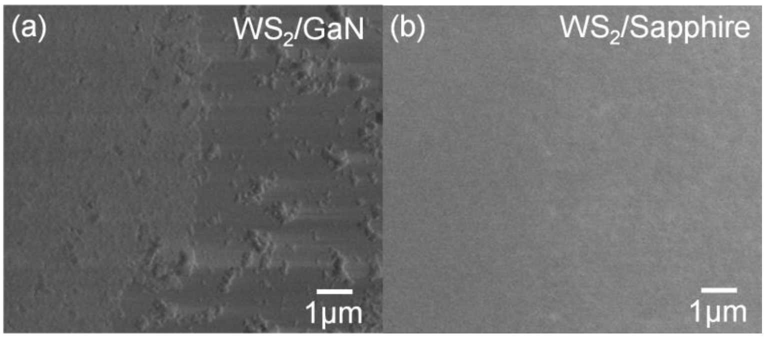

Figure S2. SEM images of different samples: (a) WS_2/GaN, and (b) WS_2/sapphire.

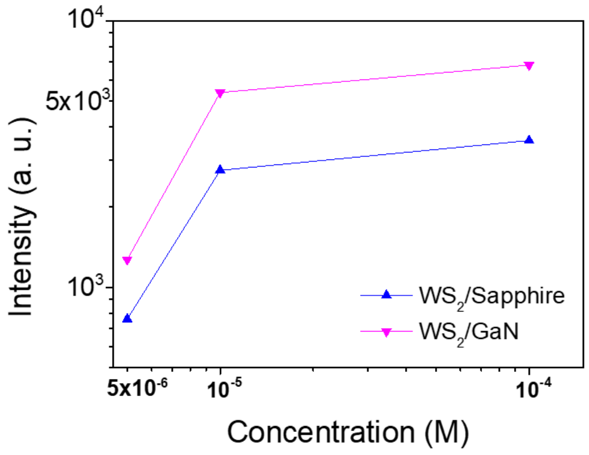

Figure S3. A relationship between the Raman peak intensities at 611 cm^{-1} and the R6G concentrations (5×10^{-6} to 10^{-4} M).
